# Molecular Dynamics Simulations and Diversity Selection by Extended Continuous Similarity Indices

**DOI:** 10.1021/acs.jcim.2c00433

**Published:** 2022-07-14

**Authors:** Anita Rácz, Levente M. Mihalovits, Dávid Bajusz, Károly Héberger, Ramón Alain Miranda-Quintana

**Affiliations:** † Plasma Chemistry Research Group, Research Centre for Natural Sciences, Magyar tudósok krt. 2, 1117 Budapest, Hungary; ‡ Medicinal Chemistry Research Group, Research Centre for Natural Sciences, Magyar tudósok krt. 2, 1117 Budapest, Hungary; § Department of Chemistry and Quantum Theory Project, University of Florida, Gainesville, Florida 32611, United States

## Abstract

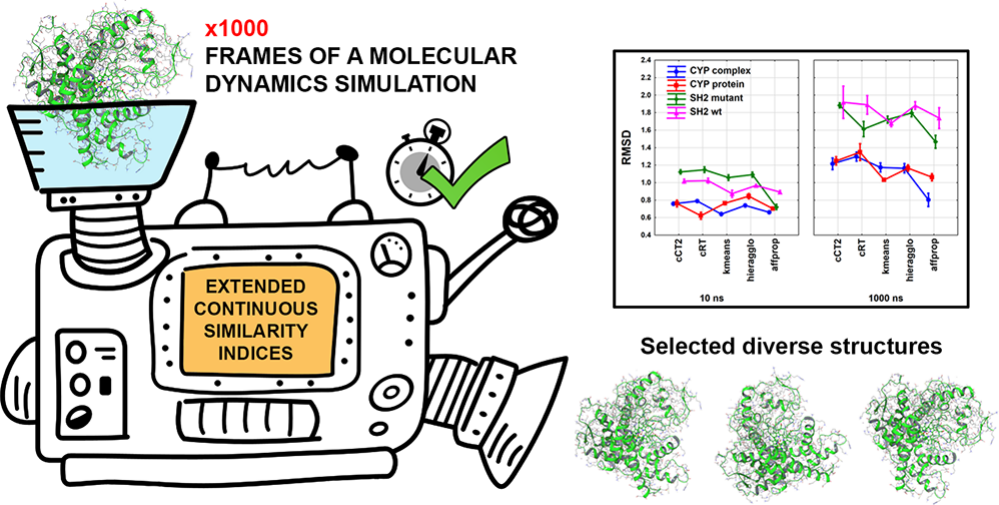

Molecular dynamics (MD) is a core methodology of molecular modeling and computational design for the study of the dynamics and temporal evolution of molecular systems. MD simulations have particularly benefited from the rapid increase of computational power that has characterized the past decades of computational chemical research, being the first method to be successfully migrated to the GPU infrastructure. While new-generation MD software is capable of delivering simulations on an ever-increasing scale, relatively less effort is invested in developing postprocessing methods that can keep up with the quickly expanding volumes of data that are being generated. Here, we introduce a new idea for sampling frames from large MD trajectories, based on the recently introduced framework of extended similarity indices. Our approach presents a new, linearly scaling alternative to the traditional approach of applying a clustering algorithm that usually scales as a quadratic function of the number of frames. When showcasing its usage on case studies with different system sizes and simulation lengths, we have registered speedups of up to 2 orders of magnitude, as compared to traditional clustering algorithms. The conformational diversity of the selected frames is also noticeably higher, which is a further advantage for certain applications, such as the selection of structural ensembles for ligand docking. The method is available open-source at https://github.com/ramirandaq/MultipleComparisons.

## 1 Introduction

With the exponential increase in computer hardware capacity, the application of molecular dynamics (MD) has become an essential tool in computational chemistry and related studies. The usage of graphical processing units (GPUs) extends the feasible length of MD simulations,^1^ allowing researchers to simulate processes even in the microsecond time scale.^2^ While many disciplines benefit from MD simulations, such as medicinal chemistry,^3^ materials science,^4^ biophysics,^5^ or biochemistry,^6^ this paper focuses mainly on the first one. MD is commonly used to examine specific events and properties on a molecular basis, most notably structural changes,^7^ structural stability,^8^ chemical reactions,^9^ and dynamics of atomic-level phenomena.^4^ Coupled with statistical thermodynamics, MD simulations are able to account for energies of simulation-related processes, as well.^10^ Additionally, structures obtained from MD trajectories aid other computational methods, too. Protein structures extracted from trajectories help to overcome the limitations of rigid ligand docking,^11^ enabling the ligands to fit into multiple protein structures.^12^ This approach is commonly termed ensemble docking^13^ and was shown to increase the performance of structure-based virtual screening, a popular method for early hit discovery in rational drug design.^14^ Moreover, the stability of these protein–ligand complexes can be verified also by MD simulations.^15^ Besides the structural data, the output trajectories of the simulations require postprocessing methods to extract valuable results. Common procedures are simulation event analysis, simulation quality analysis, and trajectory clustering.^16,17^ The latter is used frequently to obtain representative structures of the given trajectory; however, frame selection and clustering are highly nontrivial tasks, which can be heavily problem-dependent. The main questions are which indices should be used for the selection (the most frequent choice is the root mean squared distance, RMSD) and how the selection should be made (most different structures or most common structures). Commercial MD software packages (AMBERtools, Desmond, NAMD) usually contain built-in clustering programs;^16^ however, their performance is hard to measure, and their applicability for different trajectory formats is ponderous.

Therefore, as an alternative to clustering methods, we have developed a diversity picker based on the recently introduced extended continuous similarity indices, which requires only the coordinates of the atoms in the extracted snapshots of the trajectory and can be implemented easily. The algorithm is inspired by the diversity pickers commonly applied in cheminformatics to sample large chemical spaces, usually based on the use of binary molecular fingerprints.^18^ The various versions of the extended similarity indices^18−20^ have shown great promise in the problems of diversity selection^21^ and exploration of large and various data sets^22,23^ including complex biological ensembles.^24^ The keys to this success are the ability of the extended indices to quantify similarities between any number of objects and the fact that they can do so with *linear* scaling.

In ref (24), we explored the application of extended similarity indices to the *classification* of conformations in biological ensembles. To this end, we developed a novel hierarchical agglomerative clustering algorithm that successfully distinguished between conformations corresponding to different stages along multiple folding pathways. However, the fact that we had to start from a clustering step means that this approach scaled as O(*N*^2^). Moreover, we only considered extended similarity indices defined over binary vectors.^24^ That is, there was the need to perform a preprocessing step transforming the real-valued coordinates into bit-vectors via contact maps. This is problematic for three reasons: a) this preprocessing step can be time-consuming, b) it is not clear *a priori* which residues should be selected to provide an optimum contact map representation, and c) there is an intrinsic information loss when we go from real-valued to binary quantities. In this work, we overcome this latter deficiency by defining extended continuous similarity indices. Hence, a novel extended similarity-based algorithm was developed to efficiently select diverse and representative structures from long MD simulations. We evaluated the new method on case studies of MD simulations with different lengths and system sizes. The obtained trajectories were evaluated, and its performance was compared with common clustering algorithms as benchmarks. Notably, the developed algorithm was used for the postprocessing of a 100 μs long MD simulation of the SARS-CoV-2 main protease (PDB: 6Y84) to demonstrate the potential benefits of the extended continuous similarity indices, including their excellent scalability. The latter is especially relevant today, as the increase in computational capacities and access to powerful supercomputers enable access to unprecedented simulation times, but the efficiency of postprocessing methods rarely matches the capabilities of the core simulation programs. As the emphasis of this new method is on *sampling*, instead of classifying, the different conformations do not require any clustering step, which makes it very attractive, as the overall approach scales as O(*N*). Furthermore, in this manuscript, we circumvent all these issues by using a generalization of the extended similarity indices that is suitable for real-valued quantities. This means that the only preprocessing required is a simple normalization of the coordinates, that we can include as many atomic coordinates as possible, and that we are not losing any information while performing the sampling. Our method is available open-source at https://github.com/ramirandaq/MultipleComparisons.

## 2 Materials and Methods

### 2.1 Extended Continuous Similarity for MD Simulation Data

The analysis of MD data differs from the more conventional cheminformatic applications of our extended similarity indices due to the fundamentally different nature of the input data in both cases. In fact, we regard the recently introduced extended continuous similarity indices as a set of new similarity measures altogether, since they include completely original concepts to allow for the similarity calculation of an arbitrary number of continuous vectors.^20^ Nonetheless, we decided to keep the names of the existing similarity metrics that served as their basis (*e.g*., Russell-Rao, Jaccard-Tanimoto, *etc*.), so that they can be more easily traced back to the widely known, “traditional” measures. For reference, the original extended (or *n*-ary) similarity indices were defined over dichotomous variables^18,21^ (*e.g*., binary molecular fingerprints), which simplified the connection to the standard binary indices, such as the Tanimoto coefficient. However, since we are now dealing with atomic coordinates, we need to be able to efficiently process real-valued vectors. This also means that our similarity indices are capable of processing these vectors in other fields as well.^20^ The real-valued vectors in this situation demand two key aspects: first, we need to find a suitable way to normalize the coordinate values, since the extended continuous indices are defined over the [0, 1] interval. There are many (in principle, infinite) ways to perform a normalization, but the nature of this problem leads to a very natural decision. As noted above, the ultimate goal of our approach is to select different conformations that are, at the same time, as representative and diverse as possible. We also want our selection to be in accordance with standard approaches used to assess the quality of the frames selected, which is typically based on the root-mean-square distance (RMSD) values of the chosen structures. In other words, our normalization scheme must be consistent with the RMSD calculation, so the scaling procedure should not interfere with the selection algorithm. The notion of consistency^25−27^ is central to the work with similarity indices and in this particular case can be expressed quite simply: let {*q*_*i*_^(*a*)^} and {*q*_*i*_^(*b*)^} be the coordinates corresponding to any two frames, *a* and *b*, then the normalization function *n*(*q*_*i*_) must satisfy
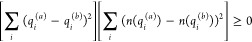
1Notice that any function that satisfies this inequality will preserve the intrinsic ordering of the conformations, so we just need to find a suitable functional form. Luckily, this can be easily done by taking
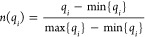
2It is critical that the minimum and maximum in the normalization function are taken over all the coordinates of all the conformations, which is the only way to guarantee a proper uniform scaling.

The second key step in the definition of the real-valued extended indices is how to obtain the analogues of the 1-similarity, 0-similarity, and dissimilarity counters introduced for the dichotomous *n*-ary indices.^18,21^ There are several variants that could establish a one-to-one correspondence between the binary and the continuous case, but once again the nature of the problem at hand suggests a simple solution. In our recent work that applied the original extended similarity indices to the study of the conformational landscape of several biomolecules,^24^ it was shown that a simple column-wise sum of the matrix of conformations was the best alternative. Hence, this will be the procedure that we will follow here, with the sum of each column of the normalized matrix taken as the central elements of the formalism.^20^

The first step is then to store the coordinates of each conformation in a row vector. Next, we arrange all these vectors in a matrix, where the rows are conformations, and the columns correspond to the coordinates of each atom. Then we proceed to normalize the entries in this matrix using eq 2 and generate a row vector containing the sum of each column of the normalized matrix. This is the key input required to calculate the extended similarity of the set of conformations. With this, we can proceed to classify the entries of the column sum vector in high-content similarity, low-content similarity, or dissimilarity counters. Finally, after appropriately weighting these counters, we can calculate any desired *n*-ary similarity index.

**Figure 1:**
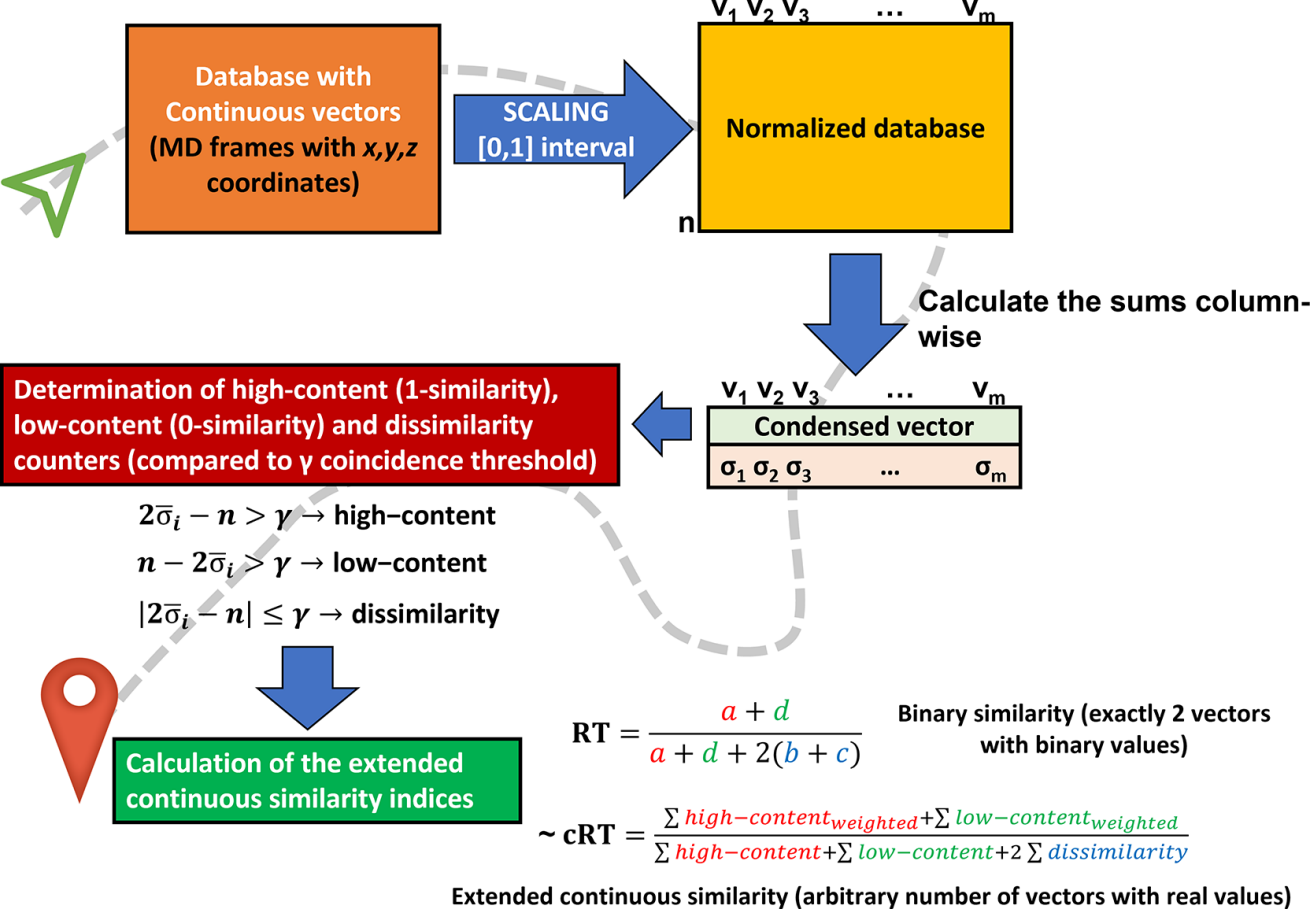
Calculation steps of the extended continuous similarity indices from a database with continuous vectors.

Figure 1 illustrates the most important steps from the starting point to the generation of extended continuous similarity indices. The details of each step can be followed in the Supporting Information with an example calculation of the nonweighted extended continuous Rogers-Tanimoto index.

### 2.2 Diversity Selection with ECS-MeDiv

The diversity selection algorithm proposed here is rooted in two central ideas previously explored in unrelated applications of the extended similarity indices: (i) medoid determination and (ii) selecting the most diverse structures from a given data set. It has been shown that the extended similarity indices provide a very attractive solution to the problem of finding the most representative element of a set (*e.g*., the medoid).^22,24^ This is done by calculating the complementary similarity of each element (that is, the extended similarity of the original set minus the corresponding element) and picking the point with lowest complementary value. The complementary similarity is a measure of how “connected” an element is to all others in the set. A “central” element (*e.g*., the medoid) will be heavily connected to the remaining elements; hence, if we remove it and calculate the similarity of the other points, this value will be low (compared to the removal of other elements). Consequently, a low complementary similarity is synonymous with the central character of an element in the set. This naturally leads to a ranking of elements, from more representative or “central” to less representative or “outliers”.

Notice, however, that this ranking is not sufficient for our present purposes. Selecting only central conformations is a bad sampling strategy because it will heavily favor native-like structures, which correspond to a very narrow region of the conformational space. Meanwhile, sampling just from the outlier structures means that we are going to miss important low-energy structures that are close to the native state. These problems cannot be solved by just using a diversity picker because we would still need to solve the problem of how to initialize the algorithm (which structure should we pick first?). That specific selection must be made reasonably and consistently, otherwise it can lead to nonreproducible errors and oversampling of outlier regions of the conformational space.

We approached these problems by combining these approaches in our *Extended Continuous Similarity – Medoid Diversity* (ECS-MeDiv) algorithm. The general strategy is as follows:

1. Select the medoid of the set as the starting conformation for the diversity picker.
2. Repeatedly, given the set of conformations already picked *C* = {*c*_1_,...,*c*_*n*_} select the conformation *c*′ such that the set {*c*_1_,...,*c*_*n*_,*c*′} has the lowest possible extended similarity.

2.1 If there are several conformations *c*′,*c*″,... that lead to the same extended similarity when added to the preselected conformations, pick the one that has the lowest value of the average binary similarity with all the elements of the preselected set.

3. Continue until the desired number of conformations has been selected (or the conformation pool has been exhausted).

This algorithm improves upon our Max_nDis picker^21^ because step 2.1 allows for a more thorough selection of the diverse conformations by effectively serving as a tiebreaker between conformations with the same extended similarity with respect to the preselected set. That is, we still use the minimization of the extended similarity (step 2) as the driving force of the algorithm, but step 2.1 adds an extra layer that leads to an even more diverse set in the end. The focus on the extended similarity also means that we do not need to generate the binary distance matrix, hence guaranteeing an efficient global exploration of all the conformations. Having to calculate a small (if any) number of binary similarities at each step implies that the ECS-MeDiv algorithm scales linearly for the selection in cases like the ones considered here, where one is only interested in a comparatively small number of conformations (5–10 out of 1000 or 100000). Notice also how by starting from the medoid (also a linearly scaling step) we ensure a uniform sampling of the MD trajectories.

### 2.3 Data Sets

**Figure 2:**
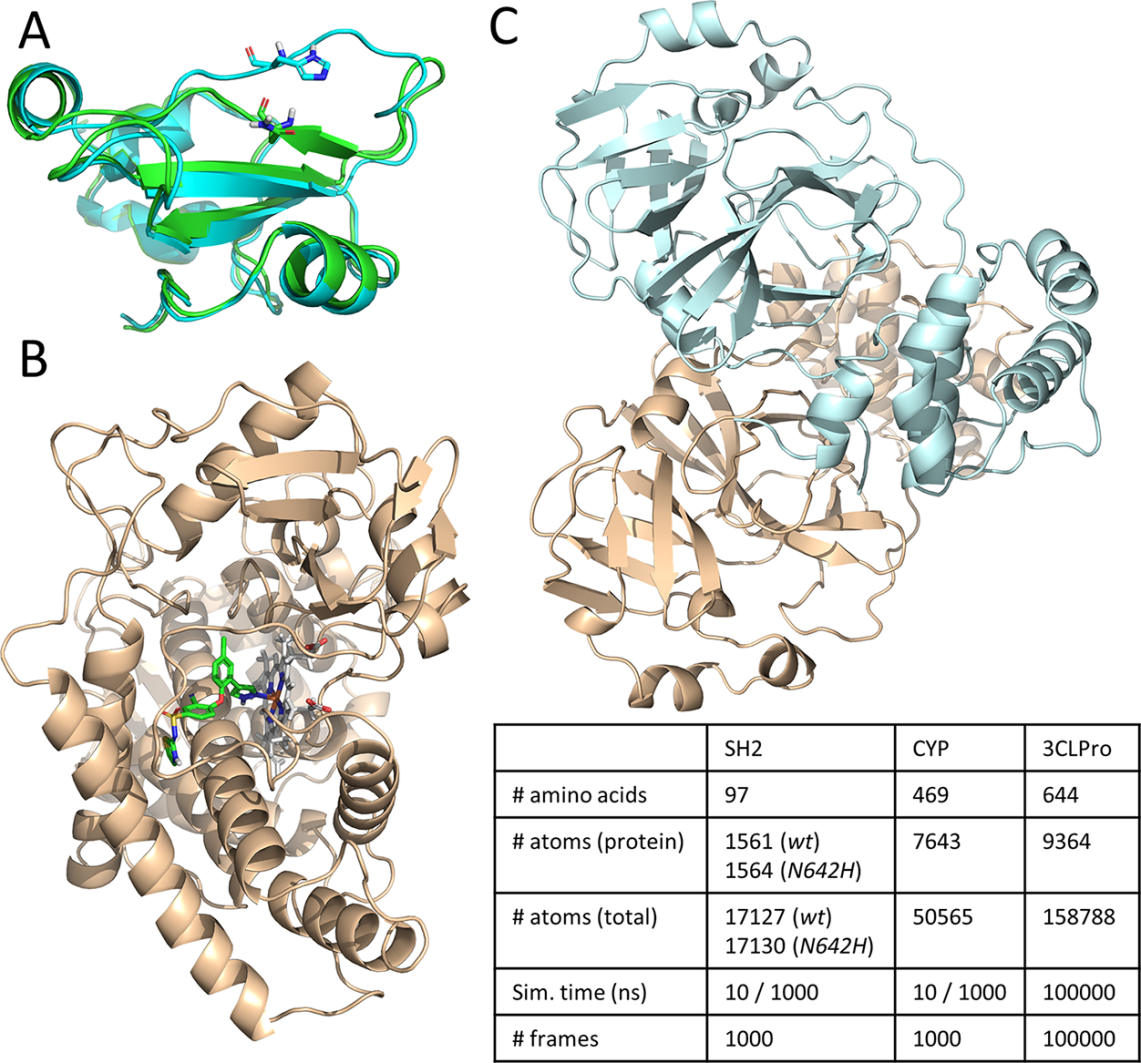
Model systems: **A)** Wild-type (green, PDB: 6MBW) and N642H mutant (cyan, PDB: 6MBZ) structure of the SH2 domain of STAT5B, with residue 642 highlighted as sticks. In the mutant structure, the uppermost β sheet is disconnected because of the mutation.^31^**B)** CYP 2C9 in complex with a small-molecule inhibitor (green sticks, PDB: 5K7K).^37^ This system was simulated in the holo (liganded, “CYP complex”) and apo (unliganded, “CYP protein”) states, and the heme cofactor (white sticks) was kept in both simulations. **C)** Dimer structure of the SARS-CoV-2 main protease in the apo (unliganded) state (PDB: 6Y84). The monomers are shown in different colors. The table contains the number of amino acids, atoms, and frames for the MD simulations of each model system.

To showcase the utility of our method, we have performed equilibrium MD simulations on two model systems (case studies) of medicinal chemistry relevance. The first system was the SH2 domain of the STAT5B transcription factor. SH2 domains are small, modular protein units that recognize phosphotyrosine-containing peptide motifs in a highly selective manner and are widely utilized in cellular signal transduction.^28^ STATs (Signal Transducers and Activators of Transcription) are a small family of multidomain proteins with pivotal roles in the regulation of DNA transcription.^29^ Dimer formation via the SH2 domain is a primary requirement of STAT function, and the inhibition of this protein–protein interaction was identified as a point of pharmaceutical intervention for several oncological indications, such as acute myeloid leukemia.^30^ This is especially relevant when STATs themselves act as oncogenesis drivers, upon point mutations in the SH2 domain.^29^ One such driver mutation of STAT5B, namely N642H, was recently identified to induce a significant conformational change in the SH2 domain.^31^ Our first model system involved the simulation of the wild-type and N642H mutant SH2 domains of STAT5B, representing two variants of a small and relatively flexible protein (Figure 2A).

The second model system is a structure of the 2C9 isoenzyme of the cytochrome P450 (CYP) protein family of heme-thiolate enzymes.^32^ CYPs have key roles in the metabolic processes of virtually all organisms by catalyzing a vast range of reactions, including the activation of molecular oxygen. In humans, hepatic CYP enzymes are the main drivers of drug metabolism,^33^ with the 2C9 isozyme being responsible for the hepatic clearance of 12–16% of clinically relevant drugs.^34^ CYP 2C9 is a highly relevant ADME (Absorption, Distribution, Metabolism, Excretion) target, and significant efforts are dedicated to the *in silico* prediction of the affinity of small molecules to this enzyme.^35,36^ Here, we have simulated the dynamics of the CYP 2C9 isozyme (PDB: 5K7K(37)) with and without its cocrystallized small-molecule inhibitor, representing a larger, more rigid system (Figure 2B).

Finally, we have demonstrated the performance of our method on a long simulation of an even larger system. To that end, we have downloaded the publicly available, 100 μs trajectory of the SARS-CoV-2 main protease performed on the Anton 2 supercomputer^38^ by D. E. Shaw Research. The main protease (3CLPro) cleaves the replicated viral polypeptides into functional proteins, and it was identified early as a potential antiviral target (Figure 2C).^39^ Since the outbreak of the COVID-19 pandemic, it was in the forefront of drug discovery efforts, from state-of-the-art crystallographic fragment screening approaches by academia^40^ to the recent breakthrough of Pfizer to provide the first approved antiviral drug specifically developed against COVID-19.^41^ Here, the simulation of the 3CLPro dimer serves as a case study to represent the current state of the art in terms of accessible simulation length on a fairly large molecular system.

### 2.4 Molecular Dynamics Simulations

Starting structures for the four proteins were extracted from the PDB structure 5K7K (CYP complex and CYP protein),^37^6MBW (SH2 wild type, wt), and 6MBZ (SH2 N642H mutant).^31^ Protein preparation was performed with Schrödinger Maestro’s Protein Preparation Wizard.^42^ For the wild-type and mutant SH2 domain structures, chains B and A were kept, respectively. The CYP complex structure contains the HEM cofactor and a bound small-molecule ligand 6RJ, while the CYP protein was created from the same PDB file with the deletion of 6RJ. System preparation was carried out with Desmond’s system builder. All structures were immersed into a minimized, buffer-sized orthorhombic TIP3P water-box. System neutralization was achieved by chlorine ion addition. The assigned force field was set to OPLS3e.^43^ The resultant solvated systems were relaxed by Desmond’s relaxation protocol. Molecular dynamics simulations were performed by Desmond in the NVT ensemble at 298.15 K, applying Nosé–Hoover temperature regulation.^44,45^ Two unbiased MD simulations were carried out for every system with simulation times of 10 and 1000 ns, respectively. These correspond to scenarios of a quick and more thorough conformational sampling by MD. Every trajectory consists of 1000 frames, which were later used for the similarity examinations and the diversity selection (clustering). The molecular dynamics simulation of the SARS-CoV-2 main protease (100 μs) was performed based on the PDB structure 6Y84 on the Anton 2 supercomputer^38^ by D. E. Shaw Research, and the trajectory was obtained from their web site: https://www.deshawresearch.com/downloads/download_trajectory_sarscov2.cgi/.

All the evaluations and diversity selection studies were based on the C, Cα, and N atoms of the protein backbone. The *x*, *y*, and *z* coordinates of these atoms were extracted with an in-house script, using VMD^46^ to convert the Desmond trajectory files into a series of mol2 format structures. The script is available at https://github.com/ramirandaq/MultipleComparisons.

### 2.5 Benchmark Clustering of the MD Simulations

The benchmark clustering was done with (i) the affinity propagation algorithm, as implemented in Schrödinger Maestro (trj_cluster.py),^47^ (ii) the hierarchical agglomerative approach,^48^ and (iii) the *k*-means method.^49^ The latter two are implemented in the cpptraj module of AMBERtools.^16^ The number of clusters was set to 5, 6, 7, 8, 9, and 10, respectively. The representative frames of the resultant clusters with the C, Cα, and N coordinates were written out for the similarity calculations. All the other parameters of the clustering methods were set to their default values.

### 2.6 Statistical Analysis

The similarity values calculated with 16 different extended continuous similarity indices were given for each system (4) and each simulation length (2). The data set consists of tables with the *x*, *y*, and *z* atomic coordinates in the columns and the actual coordinate values throughout the 1000 frames in the rows in a sequential order. The structural diversity of the MD frames was visualized with the t-distributed stochastic neighbor embedding (t-SNE) method based on the atomic coordinates.^50^ This allowed us to detect the differences of the traversed conformational space between the simulations of different lengths.

Analysis of variance (ANOVA) was used for the statistical comparison of the factors such as the (i) similarity indices (16), (ii) simulation lengths (2), or (iii) molecular systems (4). Moreover, we have analyzed the results of the diverse set selections with ANOVA, as well. STATISTICA 13 software (TIBCO) was used for the statistical analysis of the data sets. t-SNE vectors (dimensions) were calculated in the KNIME Analytics Platform,^51^ while the figures were created with GNUplot.^52^

### 2.7 RMSD Calculations

The average pairwise root-mean-square deviation (RMSD) for the given set of selected frames was calculated using an in-house script, following eqs S1 and S2. Note that RMSD refers to that average pairwise RMSD value throughout the publication. The standard deviation (std) was computed based on the difference between the average pairwise RMSD and the specific pairwise RMSDs (see eq S3).

## 3 Results and Discussion

**Figure 3:**
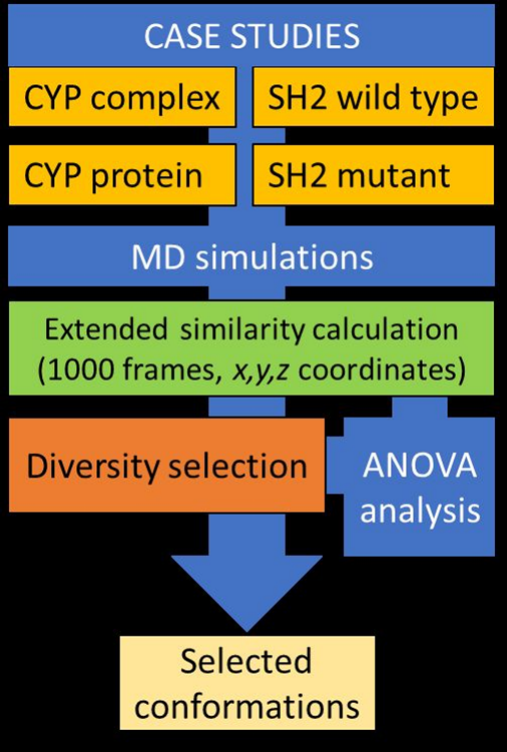
Major steps of our workflow to examine and compare the usage of various continuous similarity indices.

To explore the applicability of the proposed methodology, we examined four different biologically relevant systems (further on referred to as the CYP complex, CYP protein, SH2 wild-type, and SH2 mutant, *cf*. section 2.2, Figure 2) with 10 and 1000 ns long MD simulation lengths. The coordinates of the backbone atoms (C, Cα, and N) were extracted for the 1000 frames of each simulation, and the extended continuous similarity indices were calculated for each system and simulation length. Figure 3 shows the complete workflow of the study with the most relevant steps.

Sixteen different similarity indices were calculated for each MD run and evaluated with ANOVA. In the next step, we applied our diversity picker on the MD simulations for each case study and compared it to benchmark clustering algorithms based on the RMSD values. Finally, we showed how the extended similarity-based diversity selection protocol works on an exceptionally long MD trajectory of the main protease (3CLPro) of SARS-CoV-2 with 100,000 frames.

### 3.1 Evaluation of the MD Simulation Results

**Figure 4:**
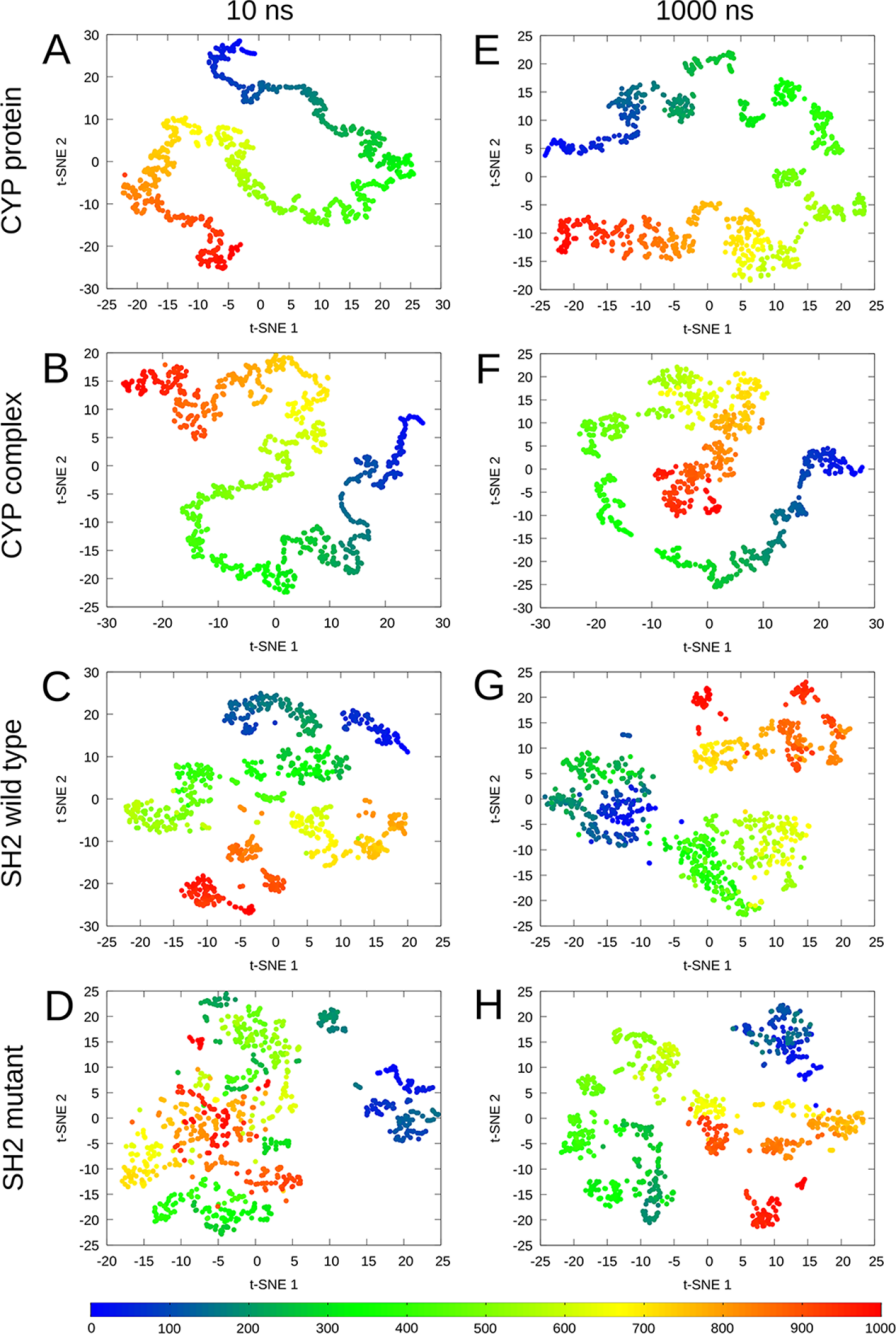
t-SNE plots of the 10 and 1000 ns long MD simulations’ coordinates of the CYP 2C9 protein (A, E), CYP 2C9-ligand complex (B, F), wild-type (C, G), and N642H mutant SH2 domains (D, H). The data points are gradually colored from blue (first frame) to red (1000th frame) following the progression of simulation time.

As a basic description of the MD simulations, we have visualized the trajectories on t-SNE plots, where the 1000 snapshots of the MD simulations, described by the *x*, *y*, and *z* coordinates, are plotted as data points on a two-dimensional graph (Figure 4). The data points are colored gradually as the simulation time progresses. This representation visualizes the sampled conformational space and gives valuable information on the heterogeneity of the MD frames during the simulation.

A simulation length of 10 ns is considered fairly short, and only slight conformational changes are expected during this period of time. However, it is common to use comparable simulation lengths for quick, equilibrium conformational sampling. Comparing the 10 ns long MDs across the different systems (CYP and SH2), the main differences arise from the size of the proteins. The SH2 domain consists of 97 residues and is much more flexible than the CYP systems with around 500 residues and an overall more rigid structure. The 10 ns t-SNE plots of the CYP systems reveal linearly progressing trajectories, in which the conformational space is discovered more sparsely than in the case of the SH2 proteins, where the frames are more scattered, especially in the case of the N642H mutant.

The 1000 ns simulation length is more suitable for examining protein dynamics, and overall, more significant conformational changes are expected in this case. The t-SNE plots of the 1000 ns simulations show similar trends as the 10 ns ones, with somewhat poorer sampling and more diffuse but still linear trajectories for the CYP systems. The degree of scattering is comparable between the ligand-bound and ligand-free systems, so the bound ligand does not seem to constrain the dynamics of the protein. Interestingly, the t-SNE plot of the wild-type SH2 domain reveals three clusters, while the mutant SH2 domain assumes several recurring conformational states.

**Figure 5:**
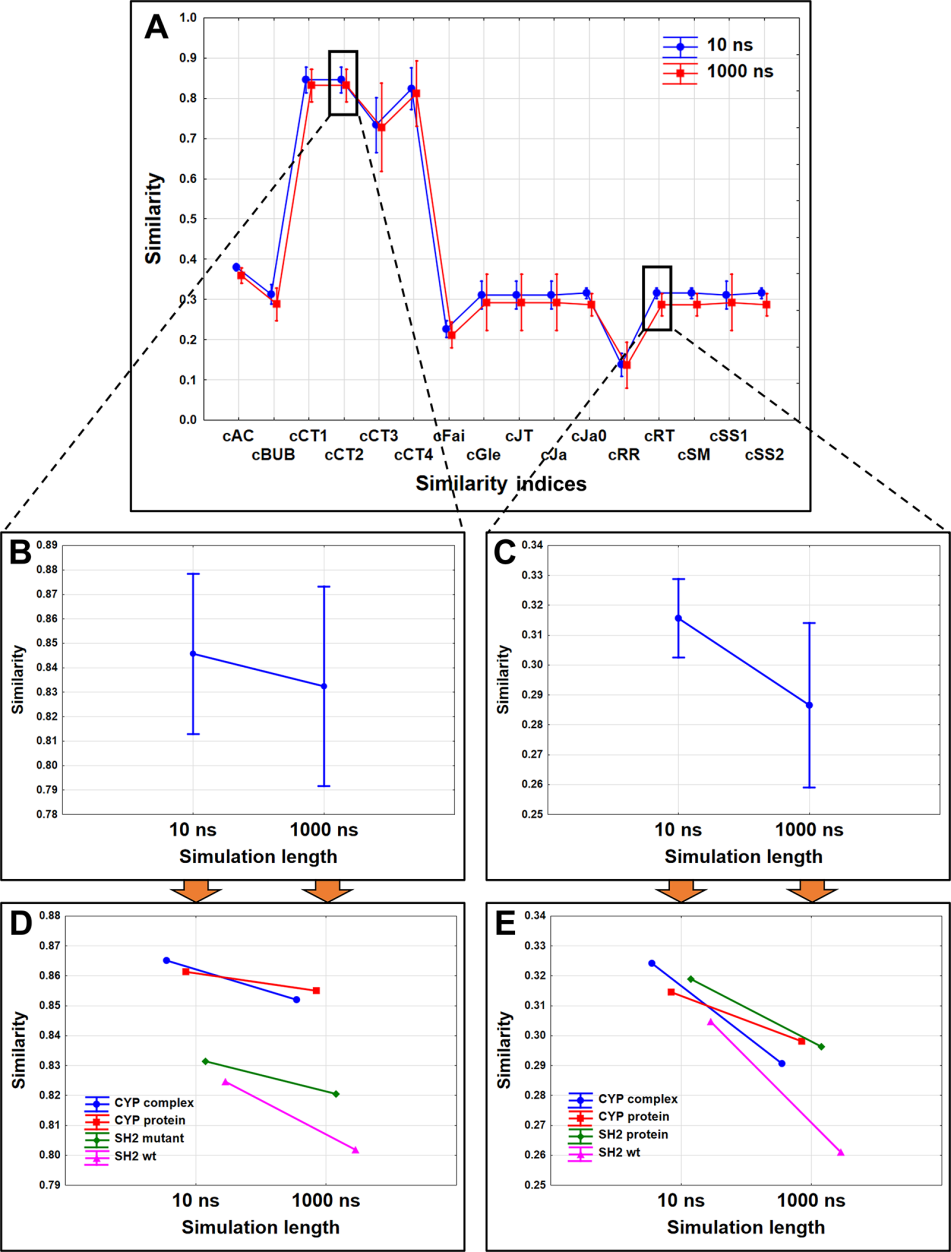
Extended continuous similarity values for the case studies merged: (A) based on the different similarity indices. cCT2 and cRT are emphasized in B and C, respectively, where the MD simulation length is compared. In the bottom row (D, E), the similarity of the frames for each case study is plotted separately in both simulation length cases. Similarities, plotted in the figure, are average similarity values with the 95% confidence intervals (except for the bottom graphs, where single similarity values are compared).

The coordinates extracted from the MD frames were used to calculate the 16 different, extended continuous similarity indices for each set of MD simulations. The similarity indices were merged into one data set, and factorial ANOVA was used for their comparison in combination with the simulation length as another factor (Figure 5). Due to the separation of the indices based on the range of the similarity values (*y* axis), we have presented the difference between the MD simulation lengths with the use of the extended continuous Consonni-Todeschini 2 and Rogers-Tanimoto (always in nonweighted versions, cCT2 and cRT, respectively) separately. As we observe the two mentioned indices more closely, the difference becomes more apparent: in both cases, the similarity between the frames is lower for the longer MD simulations, which is consistent with a more thorough sampling of the conformational space. Viewing the similarity of the case studies separately, we can see that the decrease of similarity is more pronounced for the SH2 domain simulations, especially for the wild-type protein. The result is in concordance with the findings in the t-SNE plots, where the smaller SH2 domains were shown to be more flexible than the larger CYP 2C9 protein.

### 3.2 Diversity Selection of the MD Frames

**Figure 6:**
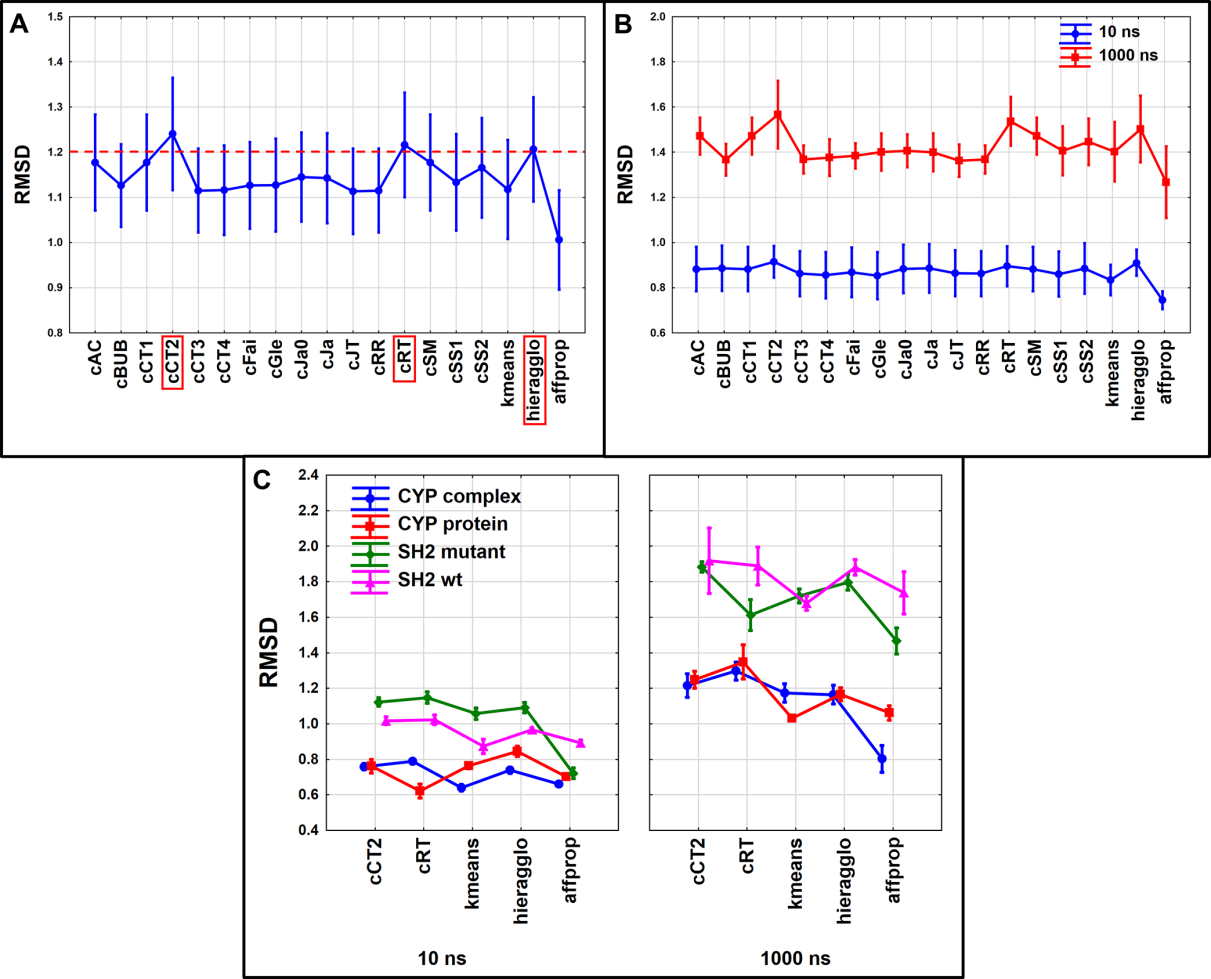
Comparison of the diversity selection methods with ANOVA. Average RMSD values of the selected frames are plotted on the *y* axis in every case. A) The red dotted line highlights the methods with the best performance (their names are highlighted on the *x* axis). B) The same results are presented with the simulation length being an additional factor in ANOVA. C) The case studies were added as a third factor for the analysis. (Only the two best indices are shown for clarity.) Average RMSD values with 95% confidence intervals are plotted in every figure.

In this section, we show the application of the extended continuous similarity indices for diversity selection, namely, to select 5 to 10 diverse frames out of each MD trajectory. This is a common practice in modeling studies, producing diverse protein conformations, *e.g*., for ensemble docking. We have compared the performance of our diversity picker to three clustering algorithms as benchmarks: (i) the affinity propagation algorithm implemented in the Schrödinger suite (from here on, affprop),^47^ (ii) the hierarchical agglomeration approach (hieragglo), and (iii) the *k*-means method (kmeans) implemented in the AMBER tools simulation package. All 16 extended continuous indices were applied for the diversity selection with the ECS-MeDiv method. In each case, we had to select the most diverse 5–10 out of the 1000 frames of trajectory. Finally, root-mean-square distance (RMSD) values were calculated between each pair of the selected frames, and the RMSD values were compared with ANOVA. The merged data set for the ANOVA comparison contained the RMSD values of each selection case (5–10 diverse frame selection), with each similarity indices (16 + 3 benchmarks) and for all the four case studies. We used RMSD values as a performance parameter because it is the most commonly used indicator in MD and 3D molecular modeling in general. Here, larger RMSDs are better, since ideally our aim is to select structurally diverse frames to better represent the complete conformational space of the protein. In terms of RMSD, two extended continuous indices, namely cCT2 and cRT, were in a comparable range with the hieragglo benchmark algorithm, although the 95% confidence intervals were very wide in each case (Figure 6A). The reason for the wide confidence intervals is clear: the range of the RMSD values is different for the 10 and 1000 ns simulation lengths (Figure 6B), with the differences between the indices being larger at the longer simulation length, as we would expect. Therefore, we have included the simulation length as a factor in the next step, along with the similarity indices and the case studies. (We have selected the most promising cCT2- and cRT-based ECS-MeDiv algorithms in Figure 6C for clarity.) All the applied factors have carried statistically significant differences (α = 0.05), and in seven out of eight cases, cCT2 or cRT could beat all three benchmark methods. Variances are established based on the data points corresponding to the selection of 5–10 frames. Interestingly, the hieragglo algorithm outperformed the other two benchmark algorithms in each case.

As an additional comparison between the extended similarity indices and the clustering algorithms, we have rank-transformed the RMSD results and counted the “wins” (highest RMSD) for each method. The cCT2-based ECS-MeDiv method dominated this competition with 12 wins, followed by the cRT-based ECS-MeDiv and the hieragglo methods with 8–8 wins each. On the other hand, cRT has the lowest sum of rankings, which means that usually the cRT-based ECS-MeDiv selection could provide higher RMSD values for the selected frames than most of the other indices (see Supporting Information Table S1).

We also compared the calculation time of our two best-performing methods and the benchmark methods on the 100 μs SARS-CoV-2 main protease trajectory as a case study for a state-of-the-art long MD simulation. The affinity propagation algorithm was omitted as it showed poor performance in the previous studies. Due to highly increased calculation times, we have compared the results only for the selection of 10 frames out of 100,000. Table 1 shows the average RMSD values between the selected 10 frames for the cRT- and cCT2-based ECS-MeDiv selections along with two benchmark methods.

**Table 1:** RMSD Values and Runtimes for Frame Selection from the 3CLPro Long MD Case Study^b^

method	RMSD	std	time (h)^a^
kmeans	1.429	0.314	35.4
hieragglo	1.395	0.270	228.0
cCT2	1.605	0.495	**3.2**
cRT	**1.701**	0.514	**3.2**

a Performed on one thread of an AMD EPYC 7451 24-Core Processor.

b std = standard deviation, see Materials and Methods.

Clearly, our extended continuous similarity-based diversity selection algorithms performed better, not only providing the best average RMSD values but also doing so in a drastically more efficient way. The calculations were 1 and 2 orders of magnitude faster than the benchmark kmeans and hieragglo algorithms, thanks to the linear (rather than quadratic) scaling of our method. Notice that while the hieragglo method came close to our algorithm in terms of RMSD values, it was the most time-demanding method.

## 4 Conclusions

We have presented the application of the extended continuous similarity indices as a novel tool to describe the conformational diversity in a set of structures, such as the frames of a molecular dynamics simulation. In addition, a new frame selection algorithm called ECS-MeDiv was developed, and its applicability and performance were demonstrated in diverse frame selection from molecular dynamics simulations and benchmarked against state-of-the-art solutions. The extended continuous similarity indices showed outstanding results in terms of RMSDs; two out of the 16, namely the cCT2 and cRT, indices could provide more diverse frame sets than the benchmark methods. Additionally, comparing the computational requirements for the frame selection from extremely long 100 μs SARS-CoV-2 main protease trajectory with 100,000 frames showed that the ECS-MeDiv, coupled with the cCT2 and cRT indices, drastically outperforms the hierarchical agglomerative method. In summary, the developed cCT2- and cRT-based ECS-MeDiv selection algorithms are suitable for the diverse selection of molecular structures extracted from MD trajectories. Moreover, the application of the extended continuous similarity indices is entirely general, and they can be easily adapted to describe the similarity of any data set with continuous values. The algorithm, along with basic usage examples, is available open-source at https://github.com/ramirandaq/MultipleComparisons.

## 5 Data and Software Availability

The Maestro molecular modeling program package and Desmond are commercial software with paid licenses. The AmberTools suite is free of charge, and its components are mostly released under the GNU General Public License (GPL). The molecular visualization software VMD is available to noncommercial users under a distribution-specific license. KNIME, the Konstanz Information Miner, is a free and open-source data analytics, reporting, and integration platform. Statistica is a proprietary advanced analytics software package. The source code of GNUplot, a portable command-line driven graphing utility, is copyrighted but freely distributed. Sample calculations and our scripts for the calculation of extended continuous similarities are available open-source at https://github.com/ramirandaq/MultipleComparisons.

## Supporting Information Available

Table of rank sums for two best extended continuous similarity indices, example calculation of nonweighted extended continuous Rogers-Tanimoto index, and table with formulas and notations for new, extended continuous similarity indices (PDF)
