# Supplementary material for: Molecular Dynamics Simulations and Diversity Selection by Extended Continuous Similarity Indices
Source: J Chem Inf Model. 2022 Jul 14;62(14):3415–25. doi: 10.1021/acs.jcim.2c00433 (PMC9326969; doi:10.1021/acs.jcim.2c00433)
Supplement: Supplementary file 1 — ci2c00433_si_001.pdf [file ci2c00433_si_001.pdf]

Supporting information

Molecular dynamics simulations and diversity selection by extended continuous similarity indices

Anita Rácz[1, ‡], *Levente M. Mihalovits*[2,‡], *Dávid Bajusz*[2],
Károly Héberger[1,*], *Ramón Alain Miranda-Quintana*[3,*]
[1] Plasma Chemistry Research Group, Research Centre for Natural Sciences, Magyar tudósok krt. 2, 1117 Budapest, Hungary
[2] Medicinal Chemistry Research Group, Research Centre for Natural Sciences, Magyar tudósok krt. 2, 1117 Budapest, Hungary
[3] Department of Chemistry and Quantum Theory Project, University of Florida, Gainesville, FL 32611, USA

[*] corresponding authors: heberger.karoly@ttk.hu, quintana@chem.ufl.edu
[‡] these authors contributed equally

Table S1. The sum of ranks and number of wins for the two best extended continuous similarity indices and the three benchmark algorithms.

	cCT2	cRT	kmeans	hieragglo	affprop
**Sum of rankings**	313	291	564	398	598
**Number of wins**	8	12	0	8	0

I. Example calculation of the non-weighted extended continuous Rogers-Tanimoto index

Let us consider the following toy model of (n = 5) real-valued vectors:

V_1	5.6584	-0.1176	4.5032	4.868	0.1256	5.7192	-0.1784	-0.2392
V_2	5.172	-0.1784	4.868	4.1384	0.308	4.6856	0.004	-0.1176
V_3	-0.2392	0.308	5.3544	0.3688	-0.0568	0.4904	0.4296	5.78
V_4	4.9896	0.0648	4.8072	5.6584	4.3816	-0.1176	-0.1176	5.0504
V_5	0.4296	-0.0568	5.2328	5.1112	-0.3	4.5032	0.612	-0.1784

The maximum and minimum values are 5.78 and -0.3, respectively.
We then proceed to normalize these entries following Eq. (2) in the manuscript:

N_1	0.98	0.03	0.79	0.85	0.07	0.99	0.02	0.01
N_2	0.9	0.02	0.85	0.73	0.1	0.82	0.05	0.03
N_3	0.01	0.1	0.93	0.11	0.04	0.13	0.12	1
N_4	0.87	0.06	0.84	0.98	0.77	0.03	0.03	0.88
N_5	0.12	0.04	0.91	0.89	0	0.79	0.15	0.02

The next step is then to calculate the sum of each column:

**σ**	2.88	0.25	4.32	3.56	0.98	2.76	0.37	1.94

Now we need to identify which of these columns correspond to high-content (hc: $2\sigma_i - n > \gamma$), low-content (lc: $n - 2\sigma_i > \gamma$), or dissimilarity (dis: $|2\sigma_i - n| \le \gamma$) counters, taking into account that, in this particular case, we take the lowest possible coincidence threshold value, $\gamma = 5 \bmod 2 = 1$. This leads to the following classification:

**counters**	dis	lc	hc	hc	lc	dis	lc	lc

Finally, we just need to calculate the weighted values of the column sums (in order to correctly penalize for partial coincidences). Here we use simple fraction weights, with $f_s(\sigma_i) = |2\sigma_i - n|/n$ for the hc or lc counters, and $f_d(\sigma_i) = 1 - (|2\sigma_i - n| - n \bmod 2)/n$ for the dis counters:

**σ**	2.88	0.25	4.32	3.56	0.98	2.76	0.37	1.94
**counters**	dis	lc	hc	hc	lc	dis	lc	lc
**w (weights)**	1.048	0.9	0.728	0.424	0.608	1.096	0.852	0.224

This is all we need to calculate the continuous extended Rogers-Tanimoto index (w_hc and w_lc stand for weighted high-content and weighted low-content, respectively):

$$cRT = \frac{\sum w \cdot hc + \sum w \cdot lc}{hc + lc + 2dis} = \frac{(0.728 + 0.424) + (0.9 + 0.608 + 0.852 + 0.224)}{2 + 4 + 2 * 2} = 0.3736 \quad (1)$$

Table S2. Summary of abbreviations, notation, and formulas corresponding to the extended continuous similarity indices.

**Additive indices**				
**Label**	**Type[a]**	**Notation[b]**	**Name**	**Equation**
cAC	cAC_hc	cACw	continuous Austin-Colwell	$s_{cAC(hc\_wd)} = \frac{2}{\pi}\arcsin\sqrt{\frac{\sum_{hc-s} f_s(\Delta_{n(k)})C_{n(k)} + \sum_{lc-s} f_s(\Delta_{n(k)})C_{n(k)}}{\sum_s f_s(\Delta_{n(k)})C_{n(k)} + \sum_d f_d(\Delta_{n(k)})C_{n(k)}}}$
		cACnw		$s_{cAC(hc\_d)} = \frac{2}{\pi}\arcsin\sqrt{\frac{\sum_{hc-s} f_s(\Delta_{n(k)})C_{n(k)} + \sum_{lc-s} f_s(\Delta_{n(k)})C_{n(k)}}{\sum_s C_{n(k)} + \sum_d C_{n(k)}}}$
cBUB	cBUB_hc	cBUBw	continuous Baroni-Urbani-Buser	$s_{cBUB(hc\_wd)} = \frac{\sqrt{\left[\sum_{hc-s} f_s(\Delta_{n(k)})C_{n(k)}\right]\left[\sum_{lc-s} f_s(\Delta_{n(k)})C_{n(k)}\right]} + \sum_{hc-s} f_s(\Delta_{n(k)})C_{n(k)}}{\left\{\sqrt{\left[\sum_{hc-s} f_s(\Delta_{n(k)})C_{n(k)}\right]\left[\sum_{lc-s} f_s(\Delta_{n(k)})C_{n(k)}\right]} + \sum_{hc-s} f_s(\Delta_{n(k)})C_{n(k)} + \sum_d f_d(\Delta_{n(k)})C_{n(k)}\right\}}$
		cBUBnw		$s_{cBUB(hc\_d)} = \frac{\sqrt{\left[\sum_{hc-s} f_s(\Delta_{n(k)})C_{n(k)}\right]\left[\sum_{lc-s} f_s(\Delta_{n(k)})C_{n(k)}\right]} + \sum_{hc-s} f_s(\Delta_{n(k)})C_{n(k)}}{\left\{\sqrt{\left[\sum_{hc-s} C_{n(k)}\right]\left[\sum_{lc-s} C_{n(k)}\right]} + \sum_{hs-s} C_{n(k)} + \sum_d C_{n(k)}\right\}}$
cCT1	cCT1_hc	cCT1w	continuous Consoni-	$s_{cCT1(hc\_wd)} = \frac{\ln\left(1 + \sum_{hc-s} f_s(\Delta_{n(k)})C_{n(k)} + \sum_{lc-s} f_s(\Delta_{n(k)})C_{n(k)}\right)}{\ln\left(1 + \sum_s f_s(\Delta_{n(k)})C_{n(k)} + \sum_d f_d(\Delta_{n(k)})C_{n(k)}\right)}$

		cCT1nw	Todeschini (1)	$s_{cCT1(hc\_d)} = \frac{\ln\left(1 + \sum_{hc-s} f_s(\Delta_{n(k)})C_{n(k)} + \sum_{lc-s} f_s(\Delta_{n(k)})C_{n(k)}\right)}{\ln\left(1 + \sum_s C_{n(k)} + \sum_d C_{n(k)}\right)}$
cCT2	cCT2_hc	cCT2w	continuous Consoni-Todeschini (2)	$s_{cCT2(hc\_wd)} = \frac{\ln\left(1 + \sum_s f_s(\Delta_{n(k)})C_{n(k)} + \sum_d f_d(\Delta_{n(k)})C_{n(k)}\right) - \ln\left(1 + \sum_d f_d(\text{[illegible]}\right.}{\ln\left(1 + \sum_s f_s(\Delta_{n(k)})C_{n(k)} + \sum_d f_d(\Delta_{n(k)})C_{n(k)}\right)}$
		cCT2nw		$s_{cCT2(hc\_d)} = \frac{\ln\left(1 + \sum_s f_s(\Delta_{n(k)})C_{n(k)} + \sum_d f_d(\Delta_{n(k)})C_{n(k)}\right) - \ln\left(1 + \sum_d f_d(\text{[illegible]}\right.}{\ln\left(1 + \sum_s C_{n(k)} + \sum_d C_{n(k)}\right)}$
cFai	cFai_hc	cFaiw	continuous Faith	$s_{cFai(hc\_wd)} = \frac{\sum_{hc-s} f_s(\Delta_{n(k)})C_{n(k)} + 0.5\sum_{lc-s} f_s(\Delta_{n(k)})C_{n(k)}}{\sum_s f_s(\Delta_{n(k)})C_{n(k)} + \sum_d f_d(\Delta_{n(k)})C_{n(k)}}$
		cFainw		$s_{cFai(hc\_d)} = \frac{\sum_{hc-s} f_s(\Delta_{n(k)})C_{n(k)} + 0.5\sum_{lc-s} f_s(\Delta_{n(k)})C_{n(k)}}{\sum_s C_{n(k)} + \sum_d C_{n(k)}}$
		cGKnw		$s_{cGK(hc\_d)} = \frac{2\min\left(\sum_{hc-s} f_s(\Delta_{n(k)})C_{n(k)}, \sum_{lc-s} f_s(\Delta_{n(k)})C_{n(k)}\right) - \sum_d f_d(\Delta_{n(k)})C_{n(k)}}{2\min\left(\sum_{hc-s} C_{n(k)}, \sum_{lc-s} C_{n(k)}\right) + \sum_d C_{n(k)}}$
		cHDnw		$s_{cHD(hc\_d)} = \frac{1}{2}\left(\frac{\sum_{hc-s} f_s(\Delta_{n(k)})C_{n(k)}}{\sum_{hc-s} C_{n(k)} + \sum_d C_{n(k)}} + \frac{\sum_{lc-s} f_s(\Delta_{n(k)})C_{n(k)}}{\sum_{lc-s} C_{n(k)} + \sum_d C_{n(k)}}\right)$
cRT	cRT_hc	cRTw	continuous Rogers-Tanimoto	$s_{cRT(hc\_wd)} = \frac{\sum_s f_s(\Delta_{n(k)})C_{n(k)}}{\sum_s f_s(\Delta_{n(k)})C_{n(k)} + 2\sum_d f_d(\Delta_{n(k)})C_{n(k)}}$
		cRTnw		$s_{cRT(hc\_d)} = \frac{\sum_s f_s(\Delta_{n(k)})C_{n(k)}}{\sum_s C_{n(k)} + 2\sum_d C_{n(k)}}$
cRG	cRG_hc	cRGw	continuous Rogot-Goldberg	$s_{cRG(hc\_wd)} = \frac{\sum_{hc-s} f_s(\Delta_{n(k)})C_{n(k)}}{2\sum_{hc-s} f_s(\Delta_{n(k)})C_{n(k)} + \sum_d f_d(\Delta_{n(k)})C_{n(k)}} + \frac{\sum_{lc-s} f_s(\Delta_{n(k)})C_{n(k)}}{2\sum_{lc-s} f_s(\Delta_{n(k)})C_{n(k)} + \sum_d f_d(\Delta_{n(k)})C_{n(k)}}$
		cRGnw		$s_{cRG(hc\_d)} = \frac{\sum_{hc-s} f_s(\Delta_{n(k)})C_{n(k)}}{2\sum_{hc-s} C_{n(k)} + \sum_d C_{n(k)}} + \frac{\sum_{lc-s} f_s(\Delta_{n(k)})C_{n(k)}}{2\sum_{lc-s} C_{n(k)} + \sum_d C_{n(k)}}$
cSM	cSM_hc	cSMw	continuous Simple matching, Sokal-Michener	$s_{cSM(hc\_wd)} = \frac{\sum_s f_s(\Delta_{n(k)})C_{n(k)}}{\sum_s f_s(\Delta_{n(k)})C_{n(k)} + \sum_d f_d(\Delta_{n(k)})C_{n(k)}}$
		cSMnw		$s_{cSM(hc\_d)} = \frac{\sum_s f_s(\Delta_{n(k)})C_{n(k)}}{\sum_s C_{n(k)} + \sum_d C_{n(k)}}$
cSS2	cSS2_hc	cSS2w	continuous Sokal-Sneath (2)	$s_{cSS2(hc\_wd)} = \frac{2\sum_s f_s(\Delta_{n(k)})C_{n(k)}}{2\sum_s f_s(\Delta_{n(k)})C_{n(k)} + \sum_d f_d(\Delta_{n(k)})C_{n(k)}}$
		cSS2nw		$s_{cSS2(hc\_wd)} = \frac{2\sum_s f_s(\Delta_{n(k)})C_{n(k)}}{2\sum_s C_{n(k)} + \sum_d C_{n(k)}}$
**Asymmetric indices**				
**Label**	**Type**	**Notation**	**Name**	**Equation**
cCT3	cCT3_hc	cCT3w	continuous Consoni-Todeschini (3)	$s_{cCT3(hc\_wd)} = \frac{\ln\left(1 + \sum_{hc-s} f_s(\Delta_{n(k)})C_{n(k)}\right)}{\ln\left(1 + \sum_s f_s(\Delta_{n(k)})C_{n(k)} + \sum_d f_d(\Delta_{n(k)})C_{n(k)}\right)}$
		cCT3nw		$s_{cCT3(hc\_d)} = \frac{\ln\left(1 + \sum_{hc-s} f_s(\Delta_{n(k)})C_{n(k)}\right)}{\ln\left(1 + \sum_s C_{n(k)} + \sum_d C_{n(k)}\right)}$
	cCT3_lc	cCT3lcw		$s_{cCT3(lc\_wd)} = \frac{\ln\left(1 + \sum_s f_s(\Delta_{n(k)})C_{n(k)}\right)}{\ln\left(1 + \sum_s f_s(\Delta_{n(k)})C_{n(k)} + \sum_d f_d(\Delta_{n(k)})C_{n(k)}\right)}$

		cCT3lcnw		$s_{cCT3(lc\_d)} = \frac{\ln\left(1+\sum_s f_s(\Delta_{n(k)})C_{n(k)}\right)}{\ln\left(1+\sum_s C_{n(k)}+\sum_d C_{n(k)}\right)}$
cCT4	cCT4_hc	cCT4w	continuous Consoni-Todeschini (4)	$s_{cCT4(hc\_wd)} = \frac{\ln\left(1+\sum_{hc-s} f_s(\Delta_{n(k)})C_{n(k)}\right)}{\ln\left(1+\sum_{hc-s} f_s(\Delta_{n(k)})C_{n(k)}+\sum_d f_d(\Delta_{n(k)})C_{n(k)}\right)}$
		cCT4nw		$s_{cCT4(hc\_d)} = \frac{\ln\left(1+\sum_{hc-s} f_s(\Delta_{n(k)})C_{n(k)}\right)}{\ln\left(1+\sum_{hc-s} C_{n(k)}+\sum_d C_{n(k)}\right)}$
	cCT4_lc	cCT4lcw		$s_{cCT4(lc\_wd)} = \frac{\ln\left(1+\sum_s f_s(\Delta_{n(k)})C_{n(k)}\right)}{\ln\left(1+\sum_s f_s(\Delta_{n(k)})C_{n(k)}+\sum_d f_d(\Delta_{n(k)})C_{n(k)}\right)}$
		cCT4lcnw		$s_{cCT4(lc\_d)} = \frac{\ln\left(1+\sum_s f_s(\Delta_{n(k)})C_{n(k)}\right)}{\ln\left(1+\sum_s C_{n(k)}+\sum_d C_{n(k)}\right)}$
cGle	cGle_hc	cGlew	continuous Gleason	$s_{cGle(hc\_wd)} = \frac{2\sum_{hc-s} f_s(\Delta_{n(k)})C_{n(k)}}{2\sum_{hc-s} f_s(\Delta_{n(k)})C_{n(k)}+\sum_d f_d(\Delta_{n(k)})C_{n(k)}}$
		cGlenw		$s_{cGle(hc\_d)} = \frac{2\sum_{hc-s} f_s(\Delta_{n(k)})C_{n(k)}}{2\sum_{hc-s} C_{n(k)}+\sum_d C_{n(k)}}$
	cGle_lc	cGlelcw		$s_{cGle(lc\_wd)} = \frac{2\sum_s f_s(\Delta_{n(k)})C_{n(k)}}{2\sum_s f_s(\Delta_{n(k)})C_{n(k)}+\sum_d f_d(\Delta_{n(k)})C_{n(k)}}$
		cGlelcnw		$s_{cGle(lc\_d)} = \frac{2\sum_s f_s(\Delta_{n(k)})C_{n(k)}}{2\sum_s C_{n(k)}+\sum_d C_{n(k)}}$
cJa	cJa_hc	cJaw	continuous Jaccard	$s_{cJa(hc\_wd)} = \frac{3\sum_{hc-s} f_s(\Delta_{n(k)})C_{n(k)}}{3\sum_{hc-s} f_s(\Delta_{n(k)})C_{n(k)}+\sum_d f_d(\Delta_{n(k)})C_{n(k)}}$
		cJanw		$s_{cJa(hc\_d)} = \frac{3\sum_{hc-s} f_s(\Delta_{n(k)})C_{n(k)}}{3\sum_{hc-s} C_{n(k)}+\sum_d C_{n(k)}}$
	cJa_lc	cJalcw		$s_{cJa(lc\_wd)} = \frac{3\sum_s f_s(\Delta_{n(k)})C_{n(k)}}{3\sum_s f_s(\Delta_{n(k)})C_{n(k)}+\sum_d f_d(\Delta_{n(k)})C_{n(k)}}$
		cJalcnw		$s_{cJa(lc\_d)} = \frac{3\sum_s f_s(\Delta_{n(k)})C_{n(k)}}{3\sum_s C_{n(k)}+\sum_d C_{n(k)}}$
cRR	cRR_hc	cRRw	continuous Russel-Rao	$s_{cRR(hc\_wd)} = \frac{\sum_{hc-s} f_s(\Delta_{n(k)})C_{n(k)}}{\sum_s f_s(\Delta_{n(k)})C_{n(k)}+\sum_d f_d(\Delta_{n(k)})C_{n(k)}}$
		cRRnw		$s_{cRR(hc\_d)} = \frac{\sum_{hc-s} f_s(\Delta_{n(k)})C_{n(k)}}{\sum_s C_{n(k)}+\sum_d C_{n(k)}}$
	cRR_lc	cRRlcw		$s_{cRR(lc\_wd)} = \frac{\sum_s f_s(\Delta_{n(k)})C_{n(k)}}{\sum_s f_s(\Delta_{n(k)})C_{n(k)}+\sum_d f_d(\Delta_{n(k)})C_{n(k)}}$
		cRRlcnw		$s_{cRR(lc\_d)} = \frac{\sum_s f_s(\Delta_{n(k)})C_{n(k)}}{\sum_s C_{n(k)}+\sum_d C_{n(k)}}$
cSS1	cSS1_hc	cSSw	continuous Sokal-Sneath (1)	$s_{cSS1(hc\_wd)} = \frac{\sum_{hc-s} f_s(\Delta_{n(k)})C_{n(k)}}{\sum_{hc-s} f_s(\Delta_{n(k)})C_{n(k)}+2\sum_d f_d(\Delta_{n(k)})C_{n(k)}}$
		cSSnw		$s_{cSS1(hc\_d)} = \frac{\sum_{hc-s} f_s(\Delta_{n(k)})C_{n(k)}}{\sum_{hc-s} C_{n(k)}+2\sum_d C_{n(k)}}$
	cSS1_lc	cSSlcw		$s_{cSS1(lc\_wd)} = \frac{\sum_s f_s(\Delta_{n(k)})C_{n(k)}}{\sum_s f_s(\Delta_{n(k)})C_{n(k)}+2\sum_d f_d(\Delta_{n(k)})C_{n(k)}}$
		cSSlcnw		$s_{cSS1(lc\_d)} = \frac{\sum_s f_s(\Delta_{n(k)})C_{n(k)}}{\sum_s C_{n(k)}+2\sum_d C_{n(k)}}$
cJT	cJT_hc	cJTw	continuous Jaccard-Tanimoto	$s_{cJT(hc\_wd)} = \frac{\sum_{hc-s} f_s(\Delta_{n(k)})C_{n(k)}}{\sum_{hc-s} f_s(\Delta_{n(k)})C_{n(k)}+\sum_d f_d(\Delta_{n(k)})C_{n(k)}}$
		cJTnw		$s_{cJT(hc\_d)} = \frac{\sum_{hc-s} f_s(\Delta_{n(k)})C_{n(k)}}{\sum_{hc-s} C_{n(k)}+\sum_d C_{n(k)}}$

	cJT_lc	cJTlcw		$s_{cJT(lc\_wd)} = \frac{\sum_s f_s(\Delta_{n(k)})C_{n(k)}}{\sum_s f_s(\Delta_{n(k)})C_{n(k)} + \sum_d f_d(\Delta_{n(k)})C_{n(k)}}$
		cJTlcnw		$s_{cJT(lc\_d)} = \frac{\sum_s f_s(\Delta_{n(k)})C_{n(k)}}{\sum_s C_{n(k)} + \sum_d C_{n(k)}}$

Equation S1. Calculation of the RMSD between two specific frames.

$$RMSD_{m,n} = \sqrt{\frac{\sum_1^t (Coord_{t,m} - Coord_{t,n})^2}{t}}$$

Where i is the number of coordinates, $Coord_{t,m}$ and $Coord_{t,n}$ denote the value of the tth coordinate of the mth and n^{th} frame, respectively. $RMSD_{m,n}$ is the specific root-mean-square deviation between the mth and n^{th} frame.

Equation S2. Calculation of the average pairwise RMSD.

$$RMSD = \frac{\sum_2^n \sum_1^{m<n} RMSD_{m,n}}{\sum_{i=1}^{n-1} i}$$

Where n is the total number of frames and RMSD is the mean pairwise root-mean-square deviation between all pairs of frames.

Equation S3. Calculation of the standard deviation (std).

$$std = \sqrt{\frac{\sum_2^n \sum_1^{m<n} (RMSD_{m,n} - RMSD)^2}{n-1}}$$

Where n is the number of frames, RMSD is the mean pairwise root mean square deviation between all pairs of frames and $RMSD_{m,n}$ is the specific root-mean-square deviation between the mth and n^{th} frame.
